# Biomarker use in predicting transcranial direct-current stimulation (tDCS) response: a systematic scoping review

**DOI:** 10.3389/fpsyt.2026.1758869

**Published:** 2026-03-18

**Authors:** Leonidas Constantinides, Anastasia Constantinidou, Andreas Chatzittofis

**Affiliations:** 1 Medical School, University of Cyprus, Nicosia, Cyprus; 2 Department of Clinical Sciences and Psychiatry, Umeå University, Umeå, Sweden

**Keywords:** biomarkers, MRI, neurology, psychiatry, tDCS, transcranial direct current stimulation, stroke, psychosis

## Abstract

**Introduction:**

Trans-cranial Direct Current Stimulation (tDCS) is a non-invasive neuromodulation technique with increasing evidence of efficacy in treating neuropsychiatric conditions. There is a need for biomarkers to predict and monitor tDCS efficacy. We aimed to conduct a systematic scoping review to assess the evidence regarding the ability of biomarkers to predict response to tDCS and identify promising candidate biomarkers in neuropsychiatric patient populations.

**Methods:**

Comprehensive searches were conducted in the MEDLINE and EMBASE databases on 24/09/2023. Articles were screened at the title and abstract level and then at full text, and inclusion/exclusion criteria applies. Data was extracted and the quality of the studies assessed with the JADAD, MINORS and Ottawa rating scales. 154 including 154 studies in the review. Disorders studied weredivided into 10 categories, Stroke/Aphasia, Psychotic Disorders, Affective Disorders, Neurocognitive Disorders, Pain Disorders, Addiction Disorders, Disorders of Consciousness, Multiple Sclerosis, Neurodevelopmental Disorders and Traumatic Brain Injury. Biomarkers with positive results in at least 2 studies in each group were identified.

**Results:**

The most common biomarker across groups was functional connectivity, appearing in 7 of 10 groups. This was followed by EEG features and Cortical Activation. The biomarkers with most evidence have plausible mechanisms of action and have been previously proposed as candidate biomarkers in similar research. Other biomarkers included anatomical and clinical features, and levels of neurotransmitters in the brain.

**Conclusion:**

This review identified the most promising candidate biomarkers in predicting and monitoring tDCS response in neuropsychiatric disorders and this may provide a focus for future research.

**Systematic review registration:**

https://osf.io/r6mkp/?view_only=64ec4811e21f43ba84fe31496f403ec2.

## 1 Introduction

Transcranial Direct Current Stimulation (tDCS) is a non-invasive neuromodulation method that applies low-intensity, persistent direct electric currents to specific cortical regions via scalp electrodes with the electrical current passing between a positively charged anode and a negatively charged cathode. It is used to modulate neuronal activity and increase synaptic plasticity with minimal side effects (1). TDCS changes the rate of baseline neuronal spontaneous depolarisation. With anodal tDCS, the depolarisation of resting membrane potential is facilitated, and neurons fire more easily. With cathodal stimulation, by hyperpolarising the resting membrane potential and the spontaneous combustion rate is lowered (2, 3). tDCS has been shown to be capable of inducing longer lasting neuroplastic changes that are clinically beneficial in the treatment of various neuropsychiatric disorders including Stroke, Traumatic Brain Injury and Depression (4–6). A recent paper using secondary meta-analysis to create evidence-based guidelines, concluded that tDCS is definitely effective (level A evidence) in treating depression, and probably effective (level B evidence) in treating a multitude of neuropsychiatric conditions such as neuropathic pain, Parkinson’s disease, stroke, schizophrenia, and alcohol addiction (7). It has potential to augment current best pharmacological treatment, but it also can be used in lieu of medications in situations where pharmacological treatment is not preferred, such as patient preference or high risk of medication side-effects (8).

Downstream effects of tDCS include neurotransmitter level changes, such as GABA changes, related to motor learning (9), and changes in the levels of monoamines, such as dopamine, which have been shown to play a role in inducing neuroplasticity (10). The effect is a change in functional connectivity in the human brain, in networks dependent on the areas targeted by tDCS treatment (11, 12). It has been shown that tDCS can induce structural plasticity, causing neurostructural changes in the brain (5).

A biomarker is defined by the European Medicines Agency as “An objective and quantifiable measure of a physiological process, pathological process or response to a treatment” (13). Biomarkers are increasingly used both in clinical practice as well as in research, where they commonly serve as endpoints in studies (14). Psychiatric and Neurological disorders are among the disorders with the highest burden of disease worldwide. In 2021, neurological disorders ranked as the leading cause of DALYs, with the largest contributors being stroke, migraine, and Alzheimer’s disease and other dementias (15). However, despite the obvious need for biomarkers in these disorders, and even though we do have a few clinically relevant biomarkers for rarer diseases, such as for fragile X syndrome, there are at present none available for common neuropsychiatric disorders like schizophrenia, bipolar disorder, and major depressive disorder (16, 17).

In the case of tDCS, the hypothesis that certain biomarkers can predict or monitor patient response has been tested by numerous studies. However, the literature has not been assessed in a systematic review. To identify the most promising patients for tDCS, to be able to predict and monitor their progress, the identification of biomarkers is a priority. Candidate biomarkers are needed if novel treatments such as TDCS are to deliver on their promise and on the promise of Precision Medicine.

There is a growing dataset of diverse studies investigating tDCS for a variety of disorders and symptoms. The heterogeneity of the literature is further compounded by the fact that tDCS is used not only for a variable number of sessions, but the sessions also themselves vary in length, and the current applied varies. Lastly, there are different forms of tDCS emerging, such as HD-tDCS. The studies themselves vary in protocol design, the use of a control group and their overall quality. Given this lack of standardization, a traditional systematic review would not be able to best highlight the output of this body of evidence. A scoping review is the most structured and systematic way to reach our objective.

Thus, the aim of this study was to examine the literature in a systematic way, assess the evidence regarding the ability of biomarkers to predict response to tDCS and identify promising candidate biomarkers in neuropsychiatric patient populations.

## 2 Methods

Database search terms were chosen to give us a wide range of useful results. We included the ‘Human’ filter in our search as we were looking for studies involving human patients. Our intervention was transcranial Direct-Current Stimulation (tDCs) and so search terms included both “transcranial direct current stimulation” and its commonly used abbreviation “tDCS”. “Biomarker” as well as related terms were searched for. Given that data from imaging, specifically MRI, is very often used as a biomarker is such studies, we also searched for MRI and related terms.

The review was registered on the Open Science Framework (osf.io) on 24/09/2023. It may be accessed at https://osf.io/r6mkp/?view_only=64ec4811e21f43ba84fe31496f403ec2.

Searches were carried out on PubMed and Embase on 24/09/2023.

Pubmed search term: “(((tdcs) OR (transcranial direct current stimulation)) OR (Transcranial Direct Current Stimulation[MeSH Terms])) AND (((((((MRI) OR (Magnetic Resonance Imaging)) OR (Magnetic Resonance Imaging[MeSH Terms])) OR (biomarkers[MeSH Terms])) OR (biomarkers)) OR (biomarker)) OR (biological marker))”. Embase search term: “Query(‘mri’/de OR mri OR ‘nuclear magnetic resonance imaging’/exp OR ‘nuclear magnetic resonance imaging’ OR ‘magnetic resonance imaging’/exp OR ‘magnetic resonance imaging’ OR ‘biomarker’/exp OR biomarker OR ‘biomarkers’/exp OR biomarkers OR ‘biological markers’/exp OR ‘biological markers’) AND (tcds OR ‘transcranial direct current stimulation’/exp OR ‘transcranial direct current stimulation’) AND ‘human’/de”. No time limits were set.

Studies were included if they were human clinical trials, if they included biomarker measurements and recorded a response to tDCS, and if they included a patient population. Studies were excluded if they did not have full text available, if they were not in English, if the clinical endpoint was not measured, if the hypothesis of the paper was not clinically relevant, if the population of the study did not include patients, and if the study included fewer than 5 patients. The review conforms to the PRISMA 2020 statement (18).

From the included articles, the following data were extracted: study title, author, year published, study type, condition studied and symptom targeted, patient number, number of male patients, patient age mean (+SD), number that underwent active tDCS, number that underwent sham tDCS, number of healthy controls, primary outcome, primary result, type of tDCS applied, duration and number of tDCS sessions, biomarker studied, biomarker results, type of biomarker. Biomarkers were classified into 4 categories: 1) Baseline, prediction of response/non-response, a biomarker measured at baseline that predicts a predefined clinical response. (In randomized clinical trials, this was further classified as 1a- general prognostic marker or 1b-a treatment-modifying biomarker, when a control arm was present). 2) Biomarkers reflecting clinical effect, 3a) Biomarkers correlated with treatment but clinical effect/change unclear/not established, 3b) Biomarkers correlated with treatment but clinical effect not achieved and 4) Biomarkers not correlated with treatment. Studies were also classified according to the characteristics of the applied tDCS protocol.

The quality of each study was assessed using structured quality assessment tools, according to the type of study. Randomised Controlled Trials were assessed using the Jadad Scale, Cohort Trials using the Newcastle-Ottawa Scale and Non-Randomised trials using the MINORS scale.

## 3 Results

**Figure 1:**
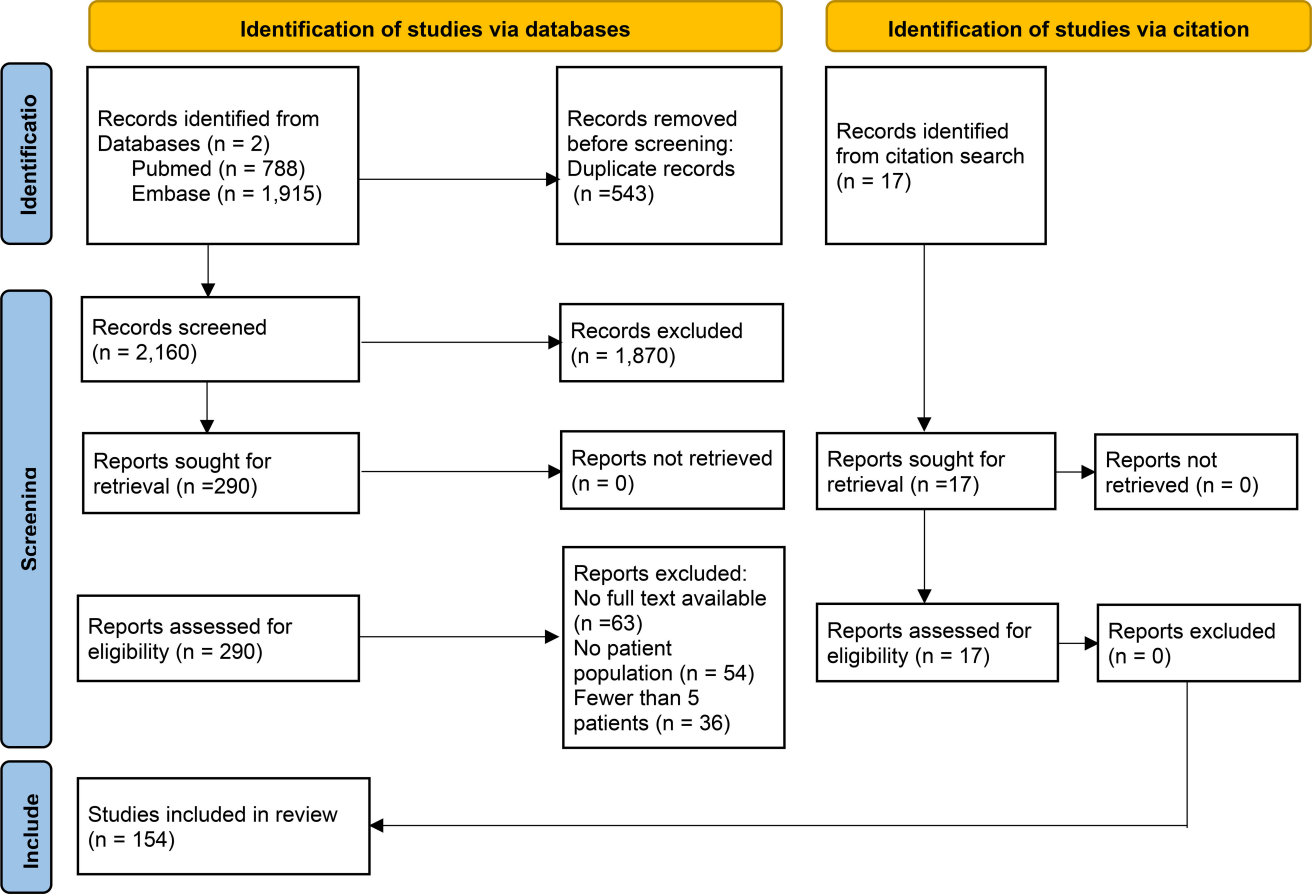
PRISMA flowchart describing the study selection process.

Search in Pubmed resulted in 788 results and Embase resulted in 1915 results respectively. After combining the results and automatically excluding duplicated results, 2196 studies were left. Meta-analyses were manually reviewed, and 17 results were added from citation searching from meta-analyses. The results were first screened for full-text review by reading the Title and Abstract. Studies that were not excluded by Title/Abstract review underwent a full-text review and the inclusion/exclusion criteria applied. 219 studies were selected for full-text review. Finally, 154 studies were included in the analysis and data extracted. The process of review can be seen in **Figure 1**.

### 3.1 Quality assessment of studies

Most studies in our review were randomized controlled trials (RCTs), which occupies the highest rank available to single studies in the hierarchy of evidence (19). 113 out of a total of 154 studies were RCTs and were graded on the Jadad scale (20) out of 5. 94 out of 113 (83%) of RCTs were graded at least 3/5, indicating a high quality of study. Non-randomized trials were assessed with the MINORS scale (21). 25 studies were non-randomized, non-controlled trials, graded out of 16 points, and 14 studies were non-randomized, controlled trials, graded out of 24 points. We designated high quality studies as achieving at least 12/16 or 18/24 on the MINORS scale. 14 out of 25 (56%) of non-controlled trials achieved at least 12/16, but only 5 out of 14 controlled trials (36%) received an assessment of at least 18/24.

2 Retrospective Studies were also identified, and their quality assessed by the Ottawa Scale (22). Both ranked 6/9, which would indicate a low-quality study.

### 3.2 Data grouping

There were 10 condition/symptom groups identified, as well as a group containing various conditions that could not be grouped with any other. The studies were divided into subgroups by condition group studied. Conditions were grouped by overall symptom similarity and the mechanisms involved in symptom production. Each study was only included in one group. The biomarkers that were found be predictive in each subgroup were found and grouped by frequency. In this way, we were able to identify the most commonly identified biomarkers that might have clinical utility in each condition group. We chose to highlight biomarkers that were identified as predictive (biomarker category 1) in at least two studies per condition group. Although an arbitrary cut-off point, this threshold was chosen in order to apply the replication criterium and be also inclusive enough to identify potential predictive biomarkers for future study. In addition, a more broadly comparable operationalization was applied for replication, and by category rather than consistent directionality and identical metric. The groups of conditions studied, the number of biomarker candidates identified and the biomarkers with positive results in two or more studies per group can be seen in **Table 1**.

**Table 1:** Overview of condition groups, studies per group, and biomarkers identified.

Condition studied	Studies	Predictive biomarkers identified in 2+ studies (Number of studies in parentheses)	Clinically reflective biomarkers identified in 2+ Studies (Number of studies in parentheses)
Stroke/Aphasia	45	Functional Connectivity (4), Cortical Activity (3), White Matter Features (3), Baseline Damage (3)	Functional Connectivity (5), Cortical Activity (5) Brain GABA levels (2)
Psychotic Disorders	18	None	Functional Connectivity (3), Cortical Activity (2)
Affective Disorders	16	None	None
Neurocognitive Disorders	17	None	Functional Connectivity (3)
Pain Disorders	13	None	Functional Connectivity (3), Glutamate/Glutamine levels in the ACC (2)
Addiction Disorders	11	None	Functional Connectivity (2)
Disorders of Consciousness	7	None	None
Multiple Sclerosis	5	None	None
Neurodevelopmental Disorders	5	None	EEG features
Traumatic Brain Injury	5	None	None
Other	12		
All Studies	154		

#### 3.2.1 Stroke/aphasia biomarkers

**Table 2** shows the data from the Stroke/Aphasia group. This group included 45 studies, 37 of them RCTs. 1045 patients were included in total. 14 biomarkers were identified as potential candidates, 4 of these were identified as being predictive biomarkers (Biomarker Type 1) in at least two different studies. These were: Functional Connectivity, Cortical activity, White matter features, Baseline Damage. Two of those biomarkers, Functional Connectivity and Cortical Activity, along with GABA concentration changes were identified as biomarkers reflecting clinical changes in at least two studies. 24 studies utilized 1mA for the tDCS protocol, 18 studies used 2mA and 3 of them 1.5mA. 31 studies set the session time at 20 minutes. 21 studies only delivered 1 session of tDCS treatment, while 24 studies delivered 10 or more sessions. The most common targets for tDCS were the inferior frontal gyrus for Aphasia studies and the primary motor cortex for motor deficit studies.

**Table 2:** Studies included in the Stroke/Aphasia group, study details and biomarkers identified.

Authors	Date	Study	Quality score	Condition	N	Age mean (SD)	tDCS current	tDCS minutes/sessions	≥10 sessions	Target area	Biomarker type	Biomarker result
Fridriksson et al. (23)	2018	RCT	5/5	Stroke - Aphasia	74	60 (10)	1mA	20mins x 15	Y	Left Temporoparietal region	1a	BDNF variant predicted response to tDCS
Marangolo et al. (24)	2014	RCT	3/5	Stroke - Aphasia	7	57.57 (6.80)	2mA	20mins x 10	Y	Ipsilesional IFG	2	tDCS resulted in BDNF level changes, related to response level
Marangolo et al. (25)	2016	RCT	3/5	Stroke - Aphasia	9	58.22 (7.10)	2mA	20mins x 15	Y	Ipsilesional IFG	2	tDCS treatment determined FC changes in lesioned hemisphere and symptom improvement
Richard et al. (26)	2020	RCT	4/5	Stroke - Cognitive Deficits	54	69.72 (7.46)	1mA	20mins x 6	N	LDLPFC	4	Brain age prediction not related to tDCS response
de Aguiar et al. (27)	2020	RCT	3/5	Primary Progressive Aphasia	30	66.4(6.72)	2mA	20mins x 10-15	Y	Left IFG	1b	Brain area volumes predicted tDCS response
Rosso et al. (28)	2014	RCT	2/5	Stroke - Aphasia	25	57(18)	1mA	15mins x 1	N	IFG	1	Broca’s Area damage predicted tDCS response
Carlson et al. (29)	2018	RCT	5/5	Stroke - Motor Deficits	15	12.1 (3.0)	1mA	20mins x 10	Y	Contralesional M1	1b	Baseline creatine/choline levels predicted tDCS response
Cipollari et al. (30)	2015	RCT	3/5	Stroke - Aphasia	6	59.17(11.09)	2mA	20mins x 10	Y	Ipsilesional IFG	2	tDCS treatment related to changes in cortical excitability, reflecting symptom improvement
Campana et al. (31)	2015	RCT	2/5	Stroke - Aphasia	20	range=37-75	2mA	20mins x 16	Y	Right IFG	1a	Left hemisphere damage predicted lower tDCS response
Bradnam et al. (32)	2011	RCT	3/5	Stroke - Motor Deficits	12	64(3.4)	1mA	20mins x 1	N	M1	1/4	FA asymmetry predicted tDCS respose, lesion size did not.
Stagg et al. (33)	2012	NRCT	01/09	Stroke - Behavioural Deficits	11	mean 64	1mA	10mins x 1	N	Ipsilesional M1	1	Cortical activity predicted tDCS response
Lee et al. (34)	2019	NRCT	17/24	Stroke - Motor Deficits	21	59.6(11.5)	2mA	20mins x 10	Y	Ipsilesional M1	1	Intrahemispheric connectivity imbalance predicted tDCS response
Hsu et al. (35)	2023	RCT	5/5	Stroke - Motor Deficits	27	59.2 (11.4)	2mA	20mins x 20	Y	Ipsilesional M1	1b	FC strength predicted response
Soliman et al. (36)	2021	RCT	3/5	Stroke - Aphasia	21	52.96(1.58)	2mA	20mins x 10	Y	Left Broca’s Area	1b	White matter changes predicted response
Cherney et al. (37)	2021	RCT	5/5	Stroke - Aphasia	12	55.54 (4.17)	1mA	13mins x 30	Y	Perilesional	2	tDCS treatment altered cortical activation reflecting clinical improvement
Cotelli et al. (38)	2016	NRCT	14/16	Primary Progressive Aphasia	18	66.5(9.5)	2mA	25mins x 10	Y	LDLPFC	1	Grey matter features predicted tDCS response
Shah-Basak et al. (39)	2020	RCT	4/5	Stroke - Aphasia	11	61.1 (12.3)	2mA	20mins x 1	N	Perilesional	2	tDCS treatment resulted in RsMEG changes, reflecting reversal of pathological abnormalities
Yang et al. (40)	2022	NRCT	18/24	Stroke - Cognitive Deficits	22	60.91(8.79)	2mA	30mins x 14	Y	LDLPFC	2	tDCS treatment altered cortical activation and FC, related to improved cognitive function scores
Lefebvre et al. (41)	2017	RCT	4/5	Stroke - Motor Deficits	22	64.7(9.8)	1mA	30mins x 1	N	Bilateral M1	2	tDCS treatment altered FC strength,related to enhancement of motor skill retention
Cheng and Schlaug (42)	2016	NRT	12/16	Stroke - Motor Deficits	5	57.4(12.9)	1.5mA	30mins x 10	Y	Motor Cortex	2	tDCS treatment resulted in resting-state FC increase, related to motor improvement
Wilmskoetter et al. (43)	2021	RCT	5/5	Stroke - Aphasia	69	59.54(10.26)	1mA	20mins x 15	Y	Temporal Lobe	1a	White matter connectivity predicted tDCS response
Yuan et al. (44)	2023	NRCT	18/24	Stroke - Sensorimotor Deficits	25	61.3(8.4)	1mA	20mins x1	N	Ipsilesional M1	1/2	tDCS treatment altered FC strength,and electric field predicted functional outcomes
Allman et al. (45)	2016	RCT	5/5	Stroke - Motor Deficits	24	62.85(11.71)	1mA	20mins x 9	N	Ipsilesional M1	2	tDCS treatment resulted in cortical activation/grey matter volume increases, reflecting clinical outcome improvement
Takeuchi et al. (46)	2012	RCT	4/5	Stroke - Motor Deficits	27	61.47(8.16)	1mA	20mins x 1	N	Ipsilesional M1	2	tDCS treatment resulted in MEP changes and improved task performance
Lee et al. (47)	2018	RCT	2/5	Stroke - Motor Deficits	24	55.4(14.8)	2mA	20mins x 10	Y	Ipsilesional M1	2	tDCS treatment altered FC strength and improved global efficiency
Lefebvre et al. (48)	2015	RCT	3/5	Stroke - Motor Deficits	19	65(10)	1mA	30mins x 1	N	Ipsilesional M1	1b	Decreased activation predicted tDCS response
Hordacre et al. (49)	2018	RCT	3/5	Stroke - Motor Deficits	10	63.8(17.9)	1mA	20mins x 2	N	Ipsilesional M1	1b	FC and lesion volume predicted tDCS response
Nair et al. (50)	2011	RCT	3/5	Stroke - Motor Deficits	14	58.5 (13.3)	1mA	30mins x 5	N	Contralesional Motor Cortex	1b	Decreased activation predicted tDCS response
O’Shea et al. (51)	2014	NRCT	16/24	Stroke - Motor Deficits	13	66.38 (13.08)	1mA	20mins x 1	N	Left M1	1	GABA levels and clinical features predicted tDCS response
Mane et al. (52)	2019	RCT	4/5	Stroke - Motor Deficits	19	34.52(19.4572)	1mA	20mins x 10	Y	Ipsilesional M1	1b	EEG features predicted poor tDCS response
Harris et al. (53)	2019	RCT	4/5	Primary Progressive Aphasia	22	66.9 (7.5)	2mA	20mins x 15	Y	LEft IFG	2	tDCS treatment resulted in decreased GABA levels and symptom improvement up to 2 months
Kolskår et al. (54)	2021	RCT	5/5	Stroke - Cognitive Deficits	54	69.13(7.37)	1mA	20mins x 6	N	LDLPFC	4	Cortical activation not related to tDCS treatment
McCambridge et al. (55)	2018	RCT	4/5	Stroke - Motor Deficits	10	69.8(6.78)	1mA	15mins x 1	N	Contralesional M1	2	tDCS treatment resulted in cortical excitability, and GABA concentration changes negatively associated with tDCS treatment response
Tao et al. (56)	2021	RCT	4/5	Primary Progressive Aphasia	32	67(6.73)	2mA	20mins x 1	N	Left IFG	3a	tDCS treatment resulted in reduced global connectivity
Licata et al. (57)	2023	RCT	3/5	Primary Progressive Aphasia	36	68.53(4.89)	2mA	20mins x 1	N	Left IFG	3a	tDCS treatment resulted in FC increase
Unger et al. (58)	2023	RCT	5/5	Stroke - Motor Deficits	17	62.58(9.77)	1mA	30mins x 30	Y	Ipsilesional M1	2	tDCS resulted in FC increase which was correlated to impairment reduction
Cunningham et al. (59)	2015	RCT	3/5	Stroke - Motor Deficits	12	61(9)	1mA	30mins x 15	Y	Ipsilesional M1	1b	Contralesional hemisphere excitability increase predicted tDCS response
Zheng and Schlaug (60)	2015	NRCT	17/24	Stroke - Motor Deficits	20	57.5(12.9)	1.5mA	30mins x 10	Y	Motor Cortex	1	FA changes predicted tDCS response
Cheng et al. (61)	2021	RCT	5/5	Stroke - Motor Deficits	18	52.89 (10.32)	1mA	20mins x 11	Y	Ipsilesional M1	1a	Baseline FC strength predicted tDCS response
Ficek et al. (62)	2018	RCT	3/5	Primary Progressive Aphasia	24	67.2 (6.5)	2mA	20mins x 15	Y	Left IFG	1b	FC changes predicted tDCS response
Lu et al. (63)	2021	RCT	1/5	Stroke - Dysphagia	60	63.39(1.75)	2mA	20mins x 30	Y	LDLPFC	1b	Brain activation volume predicted tDCS response
Nissim et al. (64)	2022	RCT	3/5	Primary Progressive Aphasia	12	66.92(6.37)	1.5mA	20mins x 1	N	Left frontotemporal region	1b	Cortical thickness and volume predicted tDCS response
Darkow et al. (65)	2017	RCT	1/5	Stroke - Aphasia	16	56.7(10.1)	1mA	20mins x 2	N	Left M1	3a	tDCS treatment resulted in functional network activity normalisation
Larcombe et al. (66)	2018	RCT	2/5	Stroke - Hemianopia	7	55.86(17.00)	1mA	20mins x 5	N	Ipsilesional Visual Cortex	3a	tDCS treatment resulted in cortical activity changes in the healthy part of the brain
Zhao et al. (67)	2021	RCT	4/5	Primary Progressive Aphasia	39	65.5(7.69)	2mA	20mins x 15	Y	Left frontal lobe	1b	White matter integrity predicted tDCS response

#### 3.2.2 Psychotic disorders biomarkers

**Table 3** shows the data from the Psychotic Disorders group. This group included 18 studies, 12 of them RCTs. 584 patients were included in total. 8 biomarkers were identified as potential candidates. However, only 3 studies identified predictive biomarkers, and none of these appeared more than once. 9 studies found that biomarker change was reflective of clinical change, and of these studies 3 identified Functional Connectivity and 2 identified Cortical Activation as potential reflective biomarkers. All but one of the studies utilized 2mA for the tDCS protocol current. 16 studies set the session time at 20 minutes, the other two at 30 minutes. 7 studies only delivered 1 or sessions of tDCS treatment, 10 studies delivered 10 sessions, and one delivered 50. The most common target for tDCS was the left dorsolateral prefrontal cortex, being a target in 13 studies.

**Table 3:** Studies included in the psychotic disorder group, study details and biomarkers identified.

Authors	Date	Study	Quality score	Condition - symptom target	N	Mean age (SD)	tDCS current	tDCS mins/sessions	≥10 sessions	Target area	Biomarker type	Biomarker result
Mondino et al. (68)	2021	Retrospective	6/9	Schizophrenia - Auditory Hallucinations	17	35.39 (8.85)	2mA	20mins x 10	Y	LDLPFC	1	Higher electric field strength predicted tDCS treatment response
Gordon et al. (69)	2019	RCT	4/5	Schizophrenia - Motor Cortical Excitability	48	34.5(8.8)	2mA	20mins x 1	N	LDLPFC	2	tDCS treatment resulted in increased intracortical inhibition as well as symptom improvement
Xu et al. (70)	2023	RCT	4/5	Schizophrenia - Cognitive function	56	40.34(9.87)	2mA	20mins x 11	Y	LDLPFC	2	HD-tDCS treatment resulted in enhanced white matter integrity, related to improvement in symptoms
Yoon et al. (71)	2019	NRT	7/16	Schizophrenia - Psychotic Symptoms	7	27.7(9)	2mA	20mins x 10	Y	LDLPFC	3a	tDCS treatment resulted in an increase in previously decreased FC
Mondino et al. (11)	2016	RCT	3/5	Schizophrenia - Auditory Verbal Hallucinations	23	37 (9.48)	2mA	20mins x 10	Y	LDLPFC	2	tDCS treatment reduced resting-state FC, related to improvement in symptoms
Impey et al. (72)	2017	RCT	5/5	Schizophrenia - Working Memory Performance	12	47.5 (12.37)	2mA	20 mins x 1	N	LDLPFC	2	tDCS treatment resulted in increased Mismatch Negativity, correlated with working memory improvement
Zhuo et al. (73)	2020	NRT	9/16	Bipolar Disorder - Auditory Hallucinations	40	40.5(11.0)	2mA	20mins x 50	Y	Left prefrontal area	2	tDCS treatments resulted in altered global FC density, reflecting a decrease in hallucination symptom severity
Kim et al. (74)	2019	RCT	5/5	Schizophrenia - Impaired Illness Awareness	11	45 (12.1)	2mA	20mins x 1	N	Biparietal/Bifrontal	3b	tDCS reduced hemispheric imbalance,increased regional cerebral blood flow, but did not change illness awareness
Dunn et al. (75)	2016	RCT	2/5	Schizophrenia - Auditory Processing Measures	36	45.07(9.86)	1mA	20mins x 1	N	LDLPFC	3a	tDCS treatment decreased Mis-Match Negativity amplitute
Marquardt et al. (76)	2022	RCT	5/5	Schizophrenia - Auditory Hallucination Neuronal Network Modulation	21	35.61(11.66)	2mA	20mins x 10	Y	LDLPFC	4	tDCS treatment had no effect in brain activation, structure or Glx/GABA levels, but small reduction in symptom severity
Palm et al. (77)	2016	RCT	4/5	Schizophrenia - Predominantly Negative Symptoms	20	36.1 (11.4)	2mA	20mins x 10	Y	LDLPFC	2	tDCS treatment resulted in FC changes and an improvement in symptom scores.
Kantrowitz et al. (78)	2019	RCT	5/5	Schizophrenia/Schizoaffective - Auditory Hallucinations	89	39.1(9.3)	2mA	20mins x 10	Y	LDLPFC	1b	Lower cognitive scores predicted better tDCS response
Orlov et al. (79)	2022	RCT	5/5	Schizophrenia - Stochastic Learning	25	36.3(9.45)	2mA	30mins x 2	N	Left medial prefrontal cortex	2	tDCS treatment resulted in altered brain activation and symptom improvement
Orlov et al. (80)	2017	RCT	5/5	Schizophrenia.- Working Memory/Executive Functions	49	35.15(7.41)	2mA	30mins x 1	N	LDLPFC	2	tDCS resulted in altered brain activation, reflected in improved symptoms
Paul et al. (81)	2022	NRT	8/16	Schizophrenia - Auditory Verbal Hallucinations	34	31.15(7.65)	2mA	20mins x 10	Y	Left TPJ	1	FC predicted responders to tDCS treatment
Kim et al. (82)	2018	NRT	12/16	Schizophrenia - Auditory Hallucinations	10	29.8 (8.4)	2mA	20mins x 1	N	LDLPFC	4	tDCS treatment did not improve P50 sensory gating to a statistically significant degree
Narita et al. (83)	2018	NRT	8/16	Schizophrenia - Psychotic Symptoms	26	40.5(10)	2mA	20mins x 10	Y	LDLPFC	2	tDCS resulted in altered oxyhaemoglobin concentrations, negatively correlated with symptom score improvement
Chang et al. (84)	2021	RCT	5/5	Schizophrenia - Insight Levels	60	44.87(10.71)	2mA	20mins x 10	Y	Prefrontal Cortex	4	tDCS treatment resulted in symptom improvement, which were not correlated to heart-rate variability

#### 3.2.3 Affective disorders biomarkers

**Table 4** shows the data from the Affective Disorders group. This group included 16studies, 12 of them RCTs. 782 patients were included in total. 11 biomarkers were identified as potential candidates. None of them emerged as predictive (type 1) or reflective (type 2) in two or more studies. 13 studies utilized 2mA for the tDCS protocol, making it by far the most popular current used for this group. 2 studies used 1.5mA and one used 1mA.15 studies set the session time at 20 or 30 minutes. Most studies delivered multiple sessions, with 14 studies delivery 10 or more. The most common target for tDCS, with 14 studies, was the left dorsolateral prefrontal cortex.

**Table 4:** Studies included in the affective disorders group, study details and biomarkers identified.

Authors	Date	Study	Quality score	Condition	N	Mean age (SD)	tDCS current	tDCS mins/sessions	≥10 sessions	Target area	Biomarker type	Biomarker result
Bulubas et al. (85)	2019	RCT	5/5	Major Depressive Disorder - Depressive Symptoms	52	40.26 (12.03)	2mA	30mins x 22	Y	LDLPFC	1b	Gray matter volume predicted tDCS antidepressant effects
Brunoni et al. (86)	2015	RCT	5/5	Major Depressive Disorder - Depressive Symptoms	73	42 (12)	2mA	30mins x 12	Y	LDLPFC	4	NT-3, NT-4, NGF and GDNF plasma levels did not significantly change with tDCS.
Brunoni et al. (87)	2014	RCT	5/5	Major Depressive Disorder - Depressive Symptoms	73	42 (12)	2mA	30mins x 13	Y	LDLPFC	4	tDCS did not significantly change BDNF levels
Zhang et al. (88)	2023	RCT	5/5	Bipolar Depression - Depressive Symptoms	50	33.06(9.00)	2mA	20mins x 14	Y	LDLPFC	2	tDCS altered Regional Homogeneity values which were correlated to improvement of symptoms
Goerigk et al. (89)	2021	RCT	5/5	Bipolar Depression - Depressive Symptoms	52	46.1 (11.06)	2mA	30mins x 10	Y	LDLPFC	1/3a	Higher plasma IL-6 predicted tDCS response and tDCS resulted in reduced IL-8
van Dam and Chrysikou. (90)	2021	RCT	2/5	Major Depressive Disorder - Emotion Regulation	19	24.11 (5.53)	1.5mA	20mins x 2	N	LDLPFC	4	tDCS did not elicit significant changes in FC
Lin et al. (91)	2021	NRT	14/16	Major Depressive Disorder/Bipolar Depression - Depressive Symptoms	80	43.27 (12.37)	2mA	20mins x 10	Y	LDLPFC	1	HR deceleration in the 1st session predicted tDCS treatment response only in unipolar depression patients
Jog et al. (92)	2021	RCT	5/5	Major Depressive Disorder - Depressive Symptoms	59	31.1(8.34)	2mA	20mins x 12	Y	LDLPFC	2	tDCS increased cerebral blood flow and improved anhedonia symptom scores
Player et al. (93)	2014	NRCT	12/24	Major Depressive Disorder - Depressive Symptoms	18	data not available	2mA	20-30mins x 1	N	LDLPFC	4	Serum BDNF levels did not change after tDCS, or correlate with change in neuroplasticity after treatment
eNord et al. (94)	2019	RCT	5/5	Major Depressive Disorder - Depressive Symptoms	39	3.38(19.97)	1mA	20mins x 8	N	Left PFC	1b	High left PFC activation predicted tDCS response
Brunoni et al. (95)	2018	RCT	4/5	Major Depressive Disorder - Depressive Symptoms	236	42.45(12.48)	2mA	30mins x 22	Y	LDLPFC	1b	NGF baseline levels predicted tDCS treatment response
Al-Kaysi et al. (96)	2017	NRT	11/16	Major Depressive Disorder - Depressive and Cognitive Symptoms	10	41.8(13.3)	2mA	20min x 15	Y	LDLPFC	1	Machine-learning EEG classification predicted tDCS response
Bulubas et al. (97)	2021	RCT	5/5	Major Depressive Disorder - Depressive Symptoms	51	40.49(11.88)	2mA	30mins x 22	Y	LDLPFC	4	Baseline rsFC did not predict tDCS response
Bersani et al. (98)	2015	NRT	9/16	Bipolar Disorder - Neurocognitive Impairments	25	45.9(12.8)	2mA	20mins x 15	Y	Right Cerebellar Cortex	3a	tDCS treatment resulted in higher amplitude and shorter latency of the P3b component
Chrysikou et al. (99)	2022	RCT	2/5	Major Depressive Disorder - Emotion Regulation	20	24.20(6.31)	1.5mA	20mins x 2	N	LDLPFC	2	tDCS treatment upregulated ventromedial PFC activity, related to performance improvement
Zanao et al. (100)	2022	RCT	5/5	Major Depressive Disorder - Depressive Symptoms	49	40.04(12.23)	2mA	30mins x 22	Y	LDLPFC	1b	Abnormalities in white matter MDD-related areas predicted tDCS antidepressant effects

#### 3.2.4 Neurocognitive disorders biomarkers

**Table 5** shows the data from the Neurocognitive Disorders group. This group included 17 studies, 14 of them RCTs. 398 patients were included in total. 13 biomarkers were identified as potential candidates. None emerged as predictive biomarkers. Functional Connectivity (identified in 3 studies) and Brain Network Configurations on fMRI (identified in 2 studies)-emerged as potential type 2 - reflective - biomarkers. 7 studies utilized 2mA for the tDCS protocol, 5 studies used 1mA and 3 of them 1.5mA. One study used slow-oscillatory tDCS, where the current oscillated sinusoidally at a frequency of 0.75 Hz. 13 studies set the session time at 20 minutes. 7 studies only delivered 1 session of tDCS treatment, 1 study delivered the most seen in our review, at 180 sessions. The most common target for tDCS was the left dorsolateral pre-frontal cortex, being a target in 8 studies.

**Table 5:** Studies included in the neurocognitive disorders group, study details and biomarkers identified.

Authors	Date	Study	Quality score	Condition	N	Mean age (SD)	tDCS current	tDCS mins/sessions	≥10 sessions	Target area	Biomarker type	Biomarker result
Pini et al. (101)	2022	RCT	5/5	AD and FTD - Cognitive Function/Behavioural Symptoms	45	71.25(8.36)	1.5mA	25mins x 10	Y	Right IPL	4	No effect of tDCS on FC or cerebral perfusion
Turnbull et al. (102)	2023	RCT	3/5	Mild Cognitive Impairment - Behavioural Symptoms	40	71 (7.0)	1.5mA	20mins x 14	Y	Left SMC	2	tDCS altered rs-FC, related to symptom improvement
Im et al. (103)	2019	RCT	4/5	Alzheimer’s Disease - Cognitive Function	18	73(7.8)	2mA	20mins x 180	Y	LDLPFC	2	Treatment preserved Cerebral Glucose metabolism and improved symptoms
Lengu et al. (104)	2021	RCT	3/5	Mild Cognitive Impairment - Neurometabolic measures	13	71.15 (5.26)	2mA	20mins x 1	N	Right SPC	3a	Treatment increased GABA and decreased the ratio of glutamate to GABA
Andrade et al. (105)	2022	RCT	5/5	Alzheimer’s Disease - Cognitive Function	36	76.3 (3.2)	2mA	30mins x 24	Y	DLPFC	2	tDCS improved symptom scores which were predictive for EEG interhemispheric coherence
Rasmussen et al. (106)	2021	RCT	5/5	Alzheimer’s Disease - Cognitive Function	19	72.58 ± 7.19	2mA	20mins x 6	N	DLPFC	2	Treatment improved symptoms scores which were correlated to fractional anisotropy
Kang et al. (107)	2021	NRT	14/16	Mild Cognitive Impairment - Cognitive Function	32	73.92(7.14)	2mA	20mins x 10	Y	Left M1	1	APOE gene, Aβ retention predicted tDCS treatment response
Emonson et al. (108)	2019	NRCT	17/24	Mild Cognitive Impairment - Cognitive Function	9	72.11(5.75)	1mA	20mins x 1	N	LDLPFC	4	tDCS did no change TMS-Evoked Potentials or Event Related Potentials in MCI patients.
Ladenbauer et al. (109)	2017	RCT	4/5	Mild Cognitive Impairment - Cognitive Function	16	71(9)	slow oscillatory-tDCS	15–25 mins x 1	N	PFC	2	so-tDCS altered EEG features, related to symptom improvement
Zhang et al. (110)	2022	RCT	2/5	Mild Cognitive Impairment - Cognitive Function	30	57.03(2.92)	1mA	20mins x 10	Y	LDLPFC	2	tDCS treatment altered brain functional network regional homogeneity (ReHo), related to clinical improvement
Vaqué-Alcázar et al. (111)	2021	RCT	5/5	Subjective Cognitive Decline - Cognitive Function	38	62.29(1.56)	1.5mA	15mins x 1	N	LDLPFC	1b	Baseline anatomical features and FC predicted treatment response
Meinzer et al. (112)	2015	RCT	2/5	Mild Cognitive Impairment - Cognitive Function	18	67.44(7.27)	1mA	20mins x 1	N	Left IFG	2	tDCS resulted in normalisation of network configuration related to improved performance
Schoellmann et al. (113)	2019	RCT	3/5	Parkinson’s Disease - Motor Symptoms	10	64.3 (11.4)	1mA	20mins x 1	N	Left SMC	2	tDCS modulated cortical activity, related to improved symptoms
Hadoush et al. (114)	2021	NRT	12/16	Parkinson’s Disease - Sleep Disturbance/Depression	25	61.48(9.19)	1mA	20mins x 10	Y	Bilateral Motor Cortex	2	tDCS resulted in reduced melatonin serum levels correlated to symptom improvement.
Pereira et al. (115)	2013	RCT	2/5	Parkinson’s Disease - Verbal Fluency network modulation	16	61.5 (9.9)	2mA	20mins x 1	N	LDLPFC/Left Parietal Cortex	2	tDCS treatment to DLPFC enhanced FC and improved performance
Conti et al. (116)	2014	RCT	4/5	HIV-associated neurocognitive disorders - Cognitive Function	11	57.9(4.5)	1.5mA	20mins x 10	Y	Cingulate cortex	2	tDCS increased FC, related to improved performance scores
Cummiford et al. (117)	2016	RCT	3/5	Mild Cognitive Impairment - Cognitive Function	22	62.91 (7.79)	2mA	20 x 8	N	Left IFG	3b	tDCS treatment increased regional resting cerebral blood flow, this was not related to clinical response

#### 3.2.5 Pain disorders biomarkers

**Table 6** shows the data from the Pain Disorders group. This group included 13 studies, 7 of them RCTs. 275 patients were included in total. 10 biomarkers were identified as potential candidates and 2 of these - Functional Connectivity and Glutamate/Glutamine levels in the ACC - were identified as type 2 biomarkers - reflecting clinical change -in at least two different studies. Each of these 2 biomarkers were also identified as predictive in a single study each, however this does not reach the cut-off threshold of two studies. 10 studies utilized 2mA for the tDCS protocol, 3 studies used 1mA. All studies set the session time at 20 minutes. 7 studies delivered 5 sessions of tDCS treatment, while 5 studies delivered 10 or more sessions. The most common target for tDCS, in 10 studies, was the primary motor cortex.

**Table 6:** Studies included in the pain disorders group, study details and biomarkers identified.

Authors	Date	Study	Quality score	Condition	N	Mean age (SD)	tDCS current	tDCS mins x sessions	≥10 sessions	Target area	Biomarker type	Biomarker result
Foerster et al. (118)	2015	NRCT	12/16	Fibromyalgia - Pain	12	47.6 (10.6)	2mA	20 x 5	N	Left Motor Cortex	1/2	Baseline Glx levels in the anterior cingulate predicted response to treatment and were lower after treatment.
Cummiford et al. (117)	2016	NRCT	13/16	Fibromyalgia - Pain	12	47.6 (10.6)	2mA	20 x 5	N	Left Motor Cortex	1/2	Stronger baseline FC predicted tDCS response, and treatment resulted in FC alterations correlated with pain reduction
Khedr et al. (119)	2017	RCT	5/5	Fibromyalgia - Pain	36	32.6 (10.99)	2mA	20 x 10	Y	Ipsilateral M1	2	Treatment resulted in changes in serum beta-endorphin levels which were correlated with symptom scale improvement
Lim et al. (120)	2021	NRCT	16/24	Fibromyalgia - Pain	12	49.3 (9.0)	2mA	20 x 5	N	Left M1	2	tDCS altered blood oxygen level dependent signals, which were correlated to pain reduction
Kikkert et al. (121)	2019	RCT	3/5	Phantom Limb - Pain	17	47 (3)	1mA	20 x 1	N	Ipsilateral S1/M1	2	Increased FC during treatment predicted phantom pain relief
Volz et al. (122)	2016	RCT	5/5	Inflammatory Bowel Disease - Pain	20	37.5 (12.9098)	2mA	20 x 5	N	Ipsilateral M1	4	Inflammatory biomarkers did not predict tDCS response
Auvichayapat et al. (123)	2018	NRT	14/16	Neuropathic Pain	10	32.7(6.88)	2mA	20 x 5	N	Left M1	2	tDCS treatment increased Glx/Cr and NAA/Cr in the ACC and improved pain
Kumru et al. (124)	2013	NRCT	20/24	Neuropathic Pain	18	49.4 (12.4)	2mA	20 x 10	Y	Contralateral M1	2	tDCS treatment reduced contact heat evoked potential amplitude, and pain perception
Pohl et al. (125)	2023	RCT	5/2	Migraine - Migraine Frequency	22	37 (13)	1mA	20 x 28	Y	Occipital Lobe	3b	tDCS resulted in reduced concentrations of GABA but not GLX or the migraine frequency
Schading et al. (126)	2021	RCT	5/2	Migraine - Migraine Frequency	24	37.80 (12.38)	1mA	20 x 28	Y	Occipital Lobe	2	tDCS application led to a reduction of migraine frequency, paralleled by grey matter volume decreases in the left lingual gyrus
Neeb et al. (127)	2019	RCT	5/5	inflammatory bowel disease - Pain	36	35.36 (12.86)	2mA	20 x 5	N	Ipsilateral M1	2	tDCS treatment increased FC and improved pain symptoms
Yoon et al. (128)	2014	NRCT	19/24	Neuropathic Pain	16	44.1 (8.6)	2mA	20 x 20	Y	Left M1	2	tDCS treatment altered cerebral glucose metabolism, related to reduced pain ratings
Suchting et al. (129)	2020	RCT	2/5	Osteoarthritis - Inflammation	40	40 (59.95)	2mA	20 x 5	N	Contralateral M1	3a	tDCS treatment resulted in lower levels of IL-6, IL-10, TNF-α, and β-endorphin

#### 3.2.6 Addiction disorders biomarkers

**Table 7** shows the data from the Addiction Disorders Group. This group included 11 studies, all but one of them RCTs. 387 patients were included in total. 6 biomarkers were identified as potential candidates and of these only Functional Connectivity was identified in at least two different studies as a potential biomarker reflecting clinical response. None of the studies in this group found, as a result, predictive biomarkers. 8 studies utilized 2mA for the tDCS protocol, 3 studies used 1mA. One study used a session time of 15 minutes, while the rest of 20 to 30 minutes. 8 studies delivered only 5 or fewer sessions of tDCS treatment, while only 3 studies delivered 10 sessions. The most common target for tDCS, in 9 studies, was the dorsolateral prefrontal cortex.

**Table 7:** Studies included in the addiction disorders group, study details and biomarkers identified.

Authors	Date	Study	Quality score	Condition	N	Mean age (SD)	tDCS current	tDCS mins x sessions	≥10 sessions	Target area	Biomarker type	Biomarker result
Kumar et al. (130)	2022	RCT	5/5	Opioid Use Disorder -Withdrawal/Craving	28	23.93(6.11)	2mA	20 x 10	Y	LDLPFC	4	Glx, GABA were not affected by HD-tDCS and were not correlated with reduction in withdrawal/craving
Conti et al. (116)	2014	NRCT	14/24	Cocaine Use Disorder - Drug-Cued Reactivity	13	30(7)	2mA	20 x 1	N	RDLPFC	3a	Prefrontal tDCS modulated the ACC response during exposure to visual drug cues in crack-cocaine users.
Holla et al. (131)	2020	RCT	5/5	Alchohol Use Disorder - Impulsivity/Time to first Lapse	21	39.00(7.31)	2mA	20 x 5	N	LDLPFC	2	tDCS resulted increased the global efficiency of brain networks, which predicted a reduced likelihood of relapse.
Mondino et al. (132)	2018	RCT	5/5	Tobacco Use Disorder - Cigarette Consumption/Craving	29	41(9.05)	2mA	20 x 10	Y	RDLPFC	2	tDCS reduced smoking craving and increased brain reactivity to smoking cues in the right posterior cingulate
Yang et al. (133)	2017	RCT	3/5	Tobacco Use Disorder - Cigarette Craving	32	26.68(6.28)	1mA	30 x 1	N	LDLPFC	2	tDCS treatment resulted in altered FC which predicted reduced craving during cue-reactivity task
Den Uyl et al. (134)	2016	RCT	4/5	Alchohol Use Disorder -Cognitive Bias	78	21.85(0.36)	1mA	15 x 3	N	LDLPFC	4	tDCS did not affect the P300 event-related potential
Dickler et al. (135)	2018	RCT	4/5	Gambling Disorder -Risk taking/Impulsivity/Craving	16	37.8(16.8)	1mA	30 x 1	N	RDLPFC	2	tDCS elevated prefrontal GABA levels, and brain metabolite levels were correlated to symptom ratings
Shahbabaie. (136)	2018	RCT	2/5	Methamphetamine use disorder - Drug Craving	15	31.33(1.40)	2mA	20 x 1	N	LDLPFC	2	tDCS modified resting-state FC which correlated to reduction of craving scores.
Sergiou et al. (137)	2022	RCT	3/5	Substance Use Disorder -Reactive Aggression	50	37.40(9.19)	2mA	20 x 10	Y	vmPFC	3b	tDCS altered some EEG features, but had no effect on trait empathy
Ekhtiari et al. (138)	2022	RCT	5/5	Methamphetamine use disorder - Drug Craving	60	35.86(8.47)	2mA	20 x 1	N	LDLPFC	3b	tDCS treatment altered FC but had no effect on craving symptoms
Nakamura - Palacios et al. (139)	2016	RCT	4/5	Substance Use Disorder - Drug Craving/Relapse	45	24.96(14.88)	2mA	20/26 x 5	N	LDLPFC	2	tDCS altered white matter parameters correlated with craving decrease, and altered ERP features on EEG

#### 3.2.7 Disorders of consciousness biomarkers

**Table 8** shows the data from the Disorders of Consciousness group. This group included 7 studies, however only 3 of them were RCTs. 126 patients were included in total. 5 biomarkers were identified as potential candidates; however, none were identified in at least two different studies as the same type of biomarker. All studies utilized 2mA and 20-minute sessions for the tDCS protocol. Four of the studies delivered only 1 session of tDCS treatment, while only 3 studies delivered 10 or more sessions. The most common target for tDCS, in 6 studies, was the left dorsolateral prefrontal cortex.

**Table 8:** Studies included in the disorders of consciousness group, study details and biomarkers identified.

Authors	Date	Study	Quality score	Condition	N	Mean age (SD)	tDCS current	tDCS mins x sessions	≥10 sessions	Target area	Biomarker type	Biomarker result
Thibaut et al. (140)	2015	retrospective	6/9	Disorders of Consciousness - Consciousness level	24	36.76 (15.65)	2mA	20 x 1	N	LDLPFC	1	Grey matter atrophy and brain hypometabolism predicted non-response to tDCS treatment.
Mensen et al. (141)	2020	NRT	7/16	Disorders of consciousness - Neural Response	7	34.7 (10.5)	2mA	20 x 1	N	LDLPFC	3b	tDCS treatment affected EEG features but did not improve outcomes.
Peng et al. (142)	2022	NRCT	19/24	Disorders of Consciousness - Consciousness level	11	43 (7.08)	2mA	20 x 10	Y	LDLPFC	2	tDCS treatment enhanced FC and clinical scores
Cavaliere et al. (143)	2016	retrospective	4/5	Disorders of Consciousness - Consciousness level	16	37.63 (14.49)	2mA	20 x 1	N	LDLPFC	1	FC features predicted response to tDCS treatment
Cai et al. (144)	2019	NRT	10/16	Disorders of Consciousness - Consciousness level	28	no data	2mA	20 x 14	Y	Parietal Region	2	tDCS treatment improved consciousness state, and in responders altered resting state EEG features
Carrière et al. (145)	2020	RCT	4/5	Disorders of Consciousness - Consciousness level	9	43.78 (13.16)	2mA	20 x 1	N	LDLPFC	3b	tDCS treatment altered hd-EEG features but did not result in symptom improvement
Zhang et al. (146)	2022	NRT	13/16	Disorders of Consciousness - Consciousness level	31	61.54(10.15)	2mA	20 x 20	Y	LDLPFC	1	The P300 ERP component predicted tDCS treatment response

#### 3.2.8 Multiple sclerosis biomarkers

**Table 9** shows the data from the Multiple Sclerosis group. This group included 5 studies, 4 of them RCTs. 73 patients were included in total. 5 biomarkers were identified as potential candidates, however none of these was identified in at least two different studies. 4 studies utilized a tDCS protocol called FaReMuS, delivering 5 15-minute sessions of tDCS at 1.5mA and targeting the S1 area.

**Table 9:** Studies included in the multiple sclerosis group, study details and biomarkers identified.

Authors	Date	Study	Quality score	Condition	N	Mean age (SD)	tDCS current	tDCS mins x sessions	≥10 sessions	Target area	Biomarker type	Biomarker result
Tecchio et al. (147)	2015	RCT	3/5	Multiple Sclerosis - Fatigue	21	42.87 (9.11)	1.5mA	15 x 5	N	Nasion-Inion line	3b	tDCS resulted in changes in S1 and M1 excitability, which did not correlate with symptom amelioration
Porcaro et al. (148)	2019	RCT	5/5	Multiple Sclerosis - Fatigue	18	44.5 (10.5)	1.5mA	15 x 5	N	S1	2	tDCS treatment normalised network connectivity and neuronal activity dynamics of S1 and M1, partly accounting for the resulting fatigue amelioration
Bertoli et al. (149)	2023	RCT	4/5	Multiple Sclerosis - Fatigue	10	35.3 (9.3)	1.5mA	15 x 5	N	S1	2	tDCS treatment induced a change in the physiological direction of the homology between the two corticospinal tracts, related to fatigue amelioration
Padalino et al. (150)	2021	NRT	11/16	Multiple Sclerosis - Fatigue	11	36 (8)	1.5mA	15 x 6	N	S1	2	tDCS treatment resulted in decrease cortico-muscular coherence, related to fatigue amelioration
Saiote et al. (151)	2014	RCT	5/5	Multiple Sclerosis - Fatigue	13	46.8 (6.8)	1mA	20 x 5	N	LDLPFC	1a	Higher lesion load predicted positive tDCS treatment response

#### 3.2.9 Neurodevelopmental disorders biomarkers

**Table 10** shows the data from the Neurodevelopmental Disorders group. This group included 5 studies, 3 of them RCTs. 92 patients were included in total. 5 biomarkers were identified as potential candidates and 1 of these -EEG features- was identified in at least two different studies as a Type 2 biomarker - reflective of clinical changes. 3 studies utilized 1mA current for the tDCS protocol, and 1 study each utilized 1.5mA and 2mA. 4 studies delivered tDCS sessions of 20 minutes duration. 3 study protocols only delivered one tDCS session, and only 1 study in this group delivered more than 5 sessions. The most common target, (in 3 studies), was the left dorsolateral pre-frontal cortex.

**Table 10:** Studies included in the neurodevelopmental disorders group, study details and biomarkers identified.

Authors	Date	Study	Quality score	Condition	N	Mean Age (SD)	tDCS current	tDCS mins x sessions	≥10 sessions	Target area	Biomarker type	Biomarker result
Auvichayapat et al. (152)	2020	NRT	12/16	Autism - Behavior	10	6.60 (0.84)	1mA	20 x 5	N	LDLPFC	2	tDCS treatment altered metabolite levels in the brain, concentration level changes of NAA/Cr, Cho/Cr, and mI/Cr were associated with improved symptom scores
Robinson - Agramonte et al. (153)	2021	NRT	12/16	Autism - Behavior	11	7.91 (1.58)	1mA	20 x 20	Y	LDLPFC	2/4	tDCS treatment resulted in reduced BDNF, no change in IGF-1, as well as improvement in clinical symptom scales
Rahimi et al. (154)	2019	RCT	3/5	Dyslexia - Temporal Resolution	17	10.35 (1.36)	1mA	20 x 1	N	Left SDG	2	tDCS treatment resulted in reduced latency and increased amplitude of auditory-evoked potentials, related to symptom score improvements.
Garnett et al. (155)	2019	RCT	4/5	Stuttering - Speech Fluency	14	22.6 (18–46)	1.5mA	20 x 1	N	SMA	3b	Stuttering severity predicted the effects of treatment on network activity, but tDCS treatment did not result in clinical improvement
Dubreuil-Vall. (156)	2021	RCT	4/5	Attention Deficit Hyperactivity Disorder - Cognitive Control	40	37.53(14.79)	2mA	30 x 1	N	LDLPFC	2	tDCS treatment modulated the P300 event-related potential as well as cognitive measures

#### 3.2.10 Traumatic brain injury biomarkers

**Table 11** Shows the data from the Traumatic Brain Injury group. This group included 5 studies, 4 of them RCTs. 144 patients were included in total. 4 biomarkers were identified as potential candidates, however none of these were identified in at least two different studies. 3 studies utilized 2mA current for the tDCS protocol. 2 studies delivered tDCS sessions of 30 minutes duration, and one study had variable duration of tDCS sessions, lasting as long as fMRI. 2 study protocols delivered 3 tDCS sessions, and 2 studies in this group delivered 10 sessions. There were varied targets for tDCS in this group, with two studies targeting the left dorsolateral pre-frontal cortex, and other studies targeting the primary motor cortex and the inferior frontal gyrus.

**Table 11:** Studies included in the traumatic brain injury group, study details and biomarkers identified.

Authors	Date	Study	Quality score	Condition	N	Mean age (SD)	tDCS current	tDCS mins x sessions	≥10 sessions	Target area	Biomarker type	Biomarker result
Quinn et al. (157	2020	RCT	4/5	Traumatic Brain Injury - Persistent Post-Traumatic Symptoms	24	33.72	2mA	30 x 10	Y	LDLPFC	3b	tDCS treatment altered cerebral blood flow, but this was not associated with neuropsychological performance and behavioural symptoms
Kurtin et al. (158)	2021	NRCT	17/24	Traumatic Brain Injury - Brain Network Activity	34	39.4(10.1)	1.8mA	18 x 3	N	Right premotor/IFG	3a	Fractional Anisotropy is correlated with brain activity changes after tDCS treatment
Wilke et al. (159)	2017	RCT	3/5	Traumatic Brain Injury - Cognition/Post-Traumatic Symptoms	17	24.2(2.8)	1mA	20 x 3	N	Left M1	4	tDCS treatment did not modulate GABA concentration and receptor activity
Quinn et al. (160)	2022	RCT	5/5	Traumatic Brain Injury - Working Memory	34	34.75(12.81)	2mA	30 x 10	Y	LDLPFC	2	tDCS treatment resulted in connectivity changes between the right DLPFC and the left anterior insula, correlated with reaction time improvement
Li et al. (161)	2019	RCT	2/5	Traumatic Brain Injury - Cognition	35	39.6(10.1)	2mA	Variable	N	Right IFG	1b	Greater post-traumatic Axonal Injury predicted worse response to tDCS treatment

## 4 Discussion

In this systematic scoping review, we identified potential biomarkers to predict response to tDCS in neuropsychiatric patient populations, as well as biomarkers reflecting clinical change.

Potential predictive biomarkers, classified as Type 1 Biomarkers in this review, were only identified in the largest group in our study, Stroke/Aphasia group. These four were Functional Connectivity, Cortical Activity, White Matter Features and Baseline Damage. The most common promising predictive biomarker in the review was functional connectivity.

The other type of biomarkers identified, were classified as Type 2, Biomarkers reflecting clinical response. We identified five potential biomarkers of this type in at least two studies across our study groups: Functional Connectivity, Cortical Activity, Brain GABA levels, Glutamate/Glutamine levels in the ACC and EEG Features. In five out of ten symptom/disorder groups, the biomarker with the most studies supporting its use reflecting response, was measures of Functional Connectivity of the brain. Our review found that as per the literature, FC is a promising predictive biomarker for tDCS response in Stroke/Aphasia, and a promising candidate as a biomarker reflecting response in Stroke/Aphasia, Psychotic Disorders, Neurocognitive Disorders, Pain Disorders and Addiction Disorders. Functional connectivity (FC) can be defined as “temporal correlations between spatially remote neurophysiological events” (162). Different functional neuroimaging techniques, such as fMRI, EEG, fNIRS etc. can be used to record signals which are then analyzed. FC can be measured at resting-state conditions, or it can be task-based. Various different analysis methods are used to estimate FC, such as: network-based functional connectivity analysis, used to delineate FC within a previously defined network of regions; resting-state functional connectivity, a time series correlation in BOLD(blood-oxygen dependent level) fMRI data acquired during the absence of an external task; and seed-based FC analysis, which delineates FC of one or more seed regions with the rest of the brain (163).

In most of the studies in this analysis, FC was defined as the statistical interdependence of BOLD signals between different regions of the brain, measured using (fMRI)). In a smaller subset of studies, other measurements such as of EEG recording features were used as to estimate FC. Specifically, fMRI has grown over the last few decades into one of the most common methods for investigating human brain function in various domains of medicine such as psychiatry and neurology (164). It is one of the most promising biomarkers in these fields, with a rapidly expanding body of evidence in the literature (165). FC has been used as a biomarker for diagnostic and patient selection purposes, such as identifying different subtypes of depression (166), to predict treatment response, such as to transcranial magnetic stimulation (167) or medications (168). In our scoping review, it surpassed any other single biomarker in the number of studies where it was of value as a biomarker, and this is to be expected given the enthusiasm with which it has been studied as a biomarker in the last years. This wide range of usefulness makes sense, given that FC can be measured for any area of the brain and therefore could theoretically be applied to areas implied in all our symptom/disorder groups. In this review it emerged as a potential biomarker that reflects response and thus could be used as a monitoring biomarker in a wide range of disorders, but also as a potential predictive biomarker in the area of Stroke/Aphasia.

EEG features were identified as a potential biomarker reflective of clinical changes in at least two studies in the group of Neurodevelopmental Disorders. It is a safe and non-invasive test, and has been used in the past to identify biomarkers in many psychiatric and neuropsychiatric disorders such as depression (169, 170), PTSD (169) and dementia (171, 172). Event-related potentials (ERPs), which are EEG changes triggered by stimuli, were specifically identified as potential biomarkers in the Neurodevelopmental Disorders group.

Cortical activation was found to be a promising predictive (type 1) biomarker in more than two studies in the Stroke/Aphasia group and a potential response (type 2) biomarker which ended up having positive results in two or more studies in the Stroke/Aphasia, and Psychotic Disorders groups. Given the functions of different parts of the cortex, the results were not surprising. The motor cortex can be a target for post-stroke movement deficits (173). Aphasia, either Primary Progressive or post-stroke, has clinical correlates in different areas of the cortex (174). In psychotic symptoms, we know that the cortex can play a significant role, such as in auditory hallucinations, where we have aberrant activation of the auditory cortex (175).

Anatomical features were identified as potential biomarkers predictive of clinical response in the Stroke/Aphasia group. They were White Matter features and Baseline damage. White matter injury in stroke is a predictive biomarker of disability (176) and of clinical outcome and white matter injury can be used as a biomarker for long-term cerebrovascular disease and dementia (177). Stroke lesion volume, grey and white matter network disruption are related to the degree of post-stroke somatosensory deficits (178).

In addition, other types of biomarkers were identified such as brain neurotransmitter levels. In the Stroke/Aphasia group, GABA levels were found to change as a result of clinical response in two studies. In fact, GABA level changes were previously identified as correlated with motor improvements after stroke (179). One of the studies in this group used GABA levels in the inferior frontal gyrus in the prefrontal cortex, an area implicated in aphasia (180). In the Pain Disorders group, two studies examined the levels of Glutamate/Glutamine in the ACC, which is involved in pain perception modulation and function abnormally in chronic pain disorders (181). Specifically, it has been shown in animal studies that glutamatergic neuronal transmission modulates pain-related aversion and contributes to chronic pain (182, 183). In humans, glutamate+glutamine concentrations at the ACC increase with the onset of pain (184).

The proposed mechanism of tDCS, i.e. increased synaptic plasticity and subsequent neurostructural brain changes, is suggestive of the potential biomarkers that might be useful in this area. Such changes in the short and long-term, are related to, and in certain studies, have been correlated to changes in the biomarkers referred to in this review, such as Functional Connectivity, Cortical activation and features of EEG. However, a large proportion of the studies included in this review delivered only a single session of tDCS and monitored biomarker changes only in the very short term, which does not fully elucidate the neurostructural changes that are the possible result of tDCS treatment.

## 5 Limitations and strengths

The main strength of this review is the systematic nature with which it was undertaken. Another strength is that the search strategy was very broad with few exclusion criteria, and a large number of results were reviewed for inclusion. It was very positive that most of the studies included were RCTs, and the large majority of those were of high quality. An important positive indicator for the candidate biomarkers is that the biomarkers identified could be connected via their mechanism of action to clinical effects and have also been proposed as biomarkers in similar research. The main weakness is the large amount of heterogeneity in the studies. We found that studies varied widely, in the tDCS parameters such as current and the number and duration of tDCS sessions delivered. Studies also differed in regard to study design, study quality and the endpoints used. In addition, the classification of Functional Connectivity as biomarkers in this review, included different modalities used in the included studies, thus adding heterogeneity in the analysis and results. This heterogeneity means that the review could not attempt a statistical meta-analysis. Another important issue is the clinical relevance of the application of biomarkers including fMRI. For biomarkers to be applicable, they such be feasible in clinical practice as well as cost-effective. The scalability of their application is also an important issue that needs to be addressed before introducing them to clinical praxis. Easily available biomarkers such as EEG and clinical measures might be more easily applicable, especially if they are already available.

## 6 Conclusion

In this systematic scoping review on biomarkers to predict and monitor tDCS results, we extracted data from 154 studies, divided by distinct groups according to disorder or symptoms studied. We identified biomarkers that had positive results in at least two studies in their study group either as predicting or reflecting clinical response. The most common biomarker with promising results overall was functional connectivity, with cortical activity the other biomarker that was found to have the most positive results predicting and monitoring tDCS response. Future tDCS research should focus on these biomarkers as they have the biggest evidence base, wide-ranging applications and plausible mechanisms of action. Research should focus on standardising tDCS protocols when it comes to current used, number and duration of sessions. As the potential mechanism of action of tDCS includes long-term, downstream neurostructural changes, the research should investigate this by delivering more sessions over a longer period and monitoring long-term biomarker changes that would reflect those downstream effects.

## Data Availability

The original contributions presented in the study are included in the article/supplementary material. Further inquiries can be directed to the corresponding author.
